# Prevalence and Genetic Diversity of a Microsporidian Parasite in the Black Imported Fire Ant and Its Social Parasitic Ant (Formicidae: Myrmicinae: *Solenopsis*) in Buenos Aires Province, Argentina

**DOI:** 10.3390/insects14120901

**Published:** 2023-11-21

**Authors:** Marina S. Ascunce, Gebreyes Kassu, Andrew Bouwma, David L. Reed, Juan Briano, David H. Oi, DeWayne Shoemaker

**Affiliations:** 1 USDA-ARS, Fire Ant Unit, Center for Medical, Agricultural, and Veterinary Entomology (CMAVE), Gainesville, FL 32608, USA; david.oi@usda.gov; 2 Florida Museum of Natural History, University of Florida, Gainesville, FL 32611, USA; gkassu@gmail.com (G.K.); dlreed@ufl.edu (D.L.R.); 3 Department of Integrative Biology, Oregon State University, Corvallis, OR 97331, USA; andrew.bouwma@oregonstate.edu; 4 Fundación para el Estudio de Especies Invasivas (FuEDEI) (ex USDA-ARS South American Biological Control Laboratory), Hurlingham, Buenos Aires B1686EFA, Argentina; jabriano@gmail.com; 5 Department of Entomology and Plant Pathology, University of Tennessee, Knoxville, TN 37996, USA; dewayne.shoemaker@utk.edu

**Keywords:** microsporidia, *Kneallhazia solenopsae*, *Thelohania solenopsae*, fire ants, social parasite, South America, SSU rRNA gene

## Abstract

**Simple Summary:**

The Black Imported Fire Ant, *Solenopsis richteri*, was accidentally introduced to the southern U.S. in the 1930s from South America. Long-term, sustained suppression approaches through biological control are needed to control growing invasive fire ant populations. Among the natural enemies used in the U.S., there have been multiple releases of a fungal-like microsporidian pathogen, *Kneallhazia solenopsae*. In this study, researchers characterized the prevalence and genetic diversity of this microsporidium in native populations of *S. richteri*. The goal of these types of studies is to assess how the natural enemy acts in the native environment to improve biological control methods. The researchers analyzed ants from 219 nests and found that the microsporidium was present in 12.8% of the nests. Interestingly, within these *S. richteri* colonies, researchers found a social parasitic ant, *Solenopsis daguerrei*, and when those ants were analyzed, 3.9% of *S. daguerrei* ants tested positive for the microsporidium. The microsporidian variants found in both *Solenopsis* species were genetically similar. Further studies are needed to evaluate the pathogenicity of this microsporidian variant.

**Abstract:**

Microsporidia are natural pathogens of arthropods and have been used as biological control against insect pests. In the United States, efforts to control the invasive Red Imported Fire Ant, *Solenopsis invicta*, and Black Imported Fire Ant, *Solenopsis richteri*, have included the use of the microsporidium, *Kneallhazia solenopsae*. However, there is limited information about the genetic differences among the microsporidian variants found in *S. invicta* and in *S. richteri*. In this study, we assessed the prevalence and genetic diversity of *K. solenopsae* in native populations of *S. richteri* in Argentina (South America). Additionally, we examined the social parasitic ant, *Solenopsis daguerrei*, which is found in some *S. richteri* nests, for the presence of this microsporidium. The survey of 219 *S. richteri* nests revealed *K. solenopsae* infections in all five sites analyzed, with 28 colonies (12.8%) positive for the microsporidium. Among the 180 *S. daguerrei* individuals collected, seven ants (3.9%) from three sites tested positive for *K. solenopsae*. Phylogenetic analyses of the microsporidian variants present in *S. richteri* and *S. daguerrei* based on partial small subunit ribosomal gene sequences (SSU rRNA) showed that both ant species shared the same variant, which is different from the ones found in *S. invicta*. Further studies are needed to determine the pathogenicity of genetically different *K. solenopsae* variants among *Solenopsis* species.

## 1. Introduction

Fire ants belong to the genus *Solenopsis*, which comprises approximately 190 species of ants [1]. Two of these species, the Black Imported Fire Ant, *S. richteri* (Forel), and the Red Imported Fire Ant, *S. invicta* (Buren), are native to South America and were accidentally introduced to the southern United States (U.S.) in the 1930s and 1940s [2,3,4]. Over the past 90 years, *S. invicta* has spread extensively, covering much of the southern U.S. and parts of California [5]. Notably, *S. invicta* has also invaded other regions of the world, such as Australia, Taiwan, China, South Korea, Japan, and the Caribbean [6,7,8]. On the other hand, invasive populations of *S. richteri* have been limited to certain areas of the southeastern U.S. where they have been interbreeding with *S. invicta*, resulting in unique hybrids only found in the U.S. [9]. Invasive fire ants are estimated to cost USD 6 billion annually in damage repair and control costs in the U.S. [10]. The affected sectors include households, electric and communications systems, agriculture, native species, and human health [10]. Integrated pest management for invasive fire ants is evolving but still mainly reliant on insecticides [11]. Long-term, sustained approaches through biological control methods are needed to suppress these growing invasive fire ant populations. Significant progress has been made in using natural enemies as biological control agents against invasive fire ants in the U.S. Among those natural enemies, there have been releases of parasitoid phorid flies in the genus *Pseudacteon*; viruses; and a fire ant microsporidian pathogen, *Kneallhazia* (=*Thelohania*) *solenopsae* Knell, Allen, and Hazard (Microsporidia: Thelohaniidae) [12].

Microsporidia are unicellular fungal parasites that have a broad impact on various animal groups, particularly insects [13]. Native populations of *S. richteri* infected with *K. solenopsae* experienced an 83% decrease in the number of active colonies [14], while *K. solenopsae* vegetative stages have been observed in all developmental stages from eggs to adult ants [15]. While the detrimental effect of *K. solenopsae* in *S. richteri* is well-documented, less is known about the genetic variability of *K. solenopsae* in *S. richteri* and whether the *K. solenopsae* infecting *S. invicta* and *S. richteri* are the same or different. Thus, a better knowledge of the genetic diversity of *K. solenopsae* is needed to improve biological control methods against fire ants.

The sequence diversity from the small subunit ribosomal gene (SSU rRNA) in *K. solenopsae* was first studied by Moser et al. [16]. They analyzed only three *K. solenopsae* isolates: one isolate from *S. invicta* in Florida (U.S.), one from Brazil, and one isolate found in *S. richteri* from Argentina. Despite the limited sampling, the authors found significant genetic variation among the different *K. solenopsae* isolates and suggested they be considered a species complex (*K. solenopsae* complex) [16]. On the other hand, cross-infection assays between species have shown that inoculations with *K. solenopsae* isolates from *S. invicta* from Florida and *S. richteri* collected in Argentina resulted in 56% fewer infections when inoculations were made to non-conspecific host colonies (Oi, unpublished data). While these reductions in infection could be attributed at least partly to poor cross-fostering of brood inocula, there is still the need to increase our knowledge of the genetic diversity of this microsporidium and the potential link to host specific pathogenesis.

To improve our understanding of the genetic diversity of *K. solenopsae* among *Solenopsis* species, Ascunce et al. [17] conducted a second study that included fire ants found in North America, namely *S. invicta*, *S. geminata* (the tropical fire ant), and *S. geminata* × *S. xyloni* hybrids, revealing an expanded host range for this microsporidium that was found in all the species analyzed [17]. Numerous *K. solenopsae* SSU rRNA variants were found that clustered in two divergent clades [17]. Furthermore, some variants were species-specific, while others were shared between *S. invicta* and native fire ants [17]. The study suggested the possibility of genetically different *K. solenopsae* species, or, at the very least, different genetic variants that seem to exhibit host preferences.

In this study we assessed the prevalence and genetic diversity of *K. solenopsae* in *S. richteri* from South America. Additionally, we included in the analysis the inquiline social parasite, *Solenopsis daguerrei* (Santschi), which was found parasitizing queens in some *S. richteri* nests. We hypothesized that the *K. solenopsae* variants in *S. richteri* would be different from the ones described in *S. invicta*. For the *K. solenopsae* variants in *S. daguerrei*, we expected those to be the same as the ones in *S. richteri* due to the intimate relationship between this inquiline social parasite and the ant host, and our previous work suggesting the potential of horizontal transmission among different *Solenopsis* species [17].

## 2. Materials and Methods

### 2.1. Sample Collection

*S. richteri*’s native distribution extends mostly in higher latitudes in South America compared with *S. invicta*’s range and includes the subtropical grasslands, Mesopotamia and Pampas. For this study, *S. richteri* worker ants were collected from 219 nests located across five geographic sites in the Province of Buenos Aires, Argentina. In 180 of those 219 *S. richteri* nests, its inquiline social parasite *S. daguerrei* was also found. In those cases, a single adult specimen (queen or male) was also assessed for the presence of the microsporidium. Ants were preserved in 95% ethanol.

### 2.2. Molecular Survey

Bulk (~10 worker ants) of *S. richteri* and individual DNA extractions of *S. daguerrei* were performed using the Puregene DNA isolation kit (Gentra Systems, Minneapolis, MI, USA). DNA concentration was measured using a spectrophotometer (Nanodrop 1000, Thermo Fisher Scientific, Wilmington, DE, USA). Polymerase chain reactions (PCR) were then used to amplify a partial region of the SSU rRNA gene to detect the presence of *K. solenopsae* following Valles et al. [18,19]. The assay is extremely sensitive and will result in successful SSU rRNA *K. solenopsae* amplification with at least one ant infected among a bulk sample of 10 workers (ratio of 1:10 infected to uninfected worker ants). All PCRs were performed as described in Ascunce et al. [17], and they were accompanied by both a known *K. solenopsae* positive control and a blank DNA-free negative control. Five μL of the PCR product were loaded onto an agarose gel and subjected to electrophoresis. The gel was stained with ethidium bromide and amplicons were visualized with UV light.

### 2.3. PCR Amplification and Sequencing

PCR amplification to obtain a larger portion of the SSU rRNA gene was conducted on eight of the *K. solenopsae*-positive samples using *K. solenopsae*-specific SSU rRNA gene primers: P933 (5′-TAGTATGTTTTGTAAGGGAGAACATAGACTATGACG-3′) and P935 (5′-ATACGGGACTATAACCCTGTATCGTGTCTGT-3′). Thermal cycling conditions for amplification reactions conducted using primers P933 and P935 were as follows: One cycle at 94 °C for 2 min, 35 cycles of 94 °C for 15 s, 59 °C for 15 s, 68 °C for 1.5 min, and a final extension cycle at 68 °C for 5 min. In this case, PCRs were conducted in 25-μL reactions containing Platinum Taq (hot start) (Invitrogen, Carlsbad, CA, USA), 0.4 μM of each primer, 0.5-to-1 μL of total genomic DNA (25 to 50 ng) and water. PCR amplicons were purified using magnetic beads (Agencourt AMPure, Beverly, MA, USA) and used in standard fluorescent cycle-sequencing PCR reactions (ABI Prism BigDye TM Terminator chemistry, Applied Biosystems, Foster City, CA, USA). Sequencing reactions were purified using Agencourt CleanSEQ (Beverly, MA, USA) magnetic beads and run on an automatic sequencer at the sequencing core facility (ICBR) at the University of Florida.

### 2.4. Phylogenetic Analysis

Nucleotide sequences were edited using Geneious Prime Ver. 2022.0.2 (Biomatters, Inc., Boston, MA, USA). The resulting sequences were combined with previously published SSU rRNA sequences of *K. solenopsae* from *S. rictheri* host (GenBank Accession number: AF031537, Moser et al. [16]), classified as Genotype SA_1 per Ascunce et al. [17]; *K. solenopsae* from *S. invicta* host from South America (GenBank Accession number: AF031538, Moser et al. [16]), classified as Genotype SA_2 per Ascunce et al. [17]; and *K. solenopsae* from *S. invicta* host in the U.S. (GenBank Accession number: AF134205, Moser et al. [16]), classified as Genotype WD_1 per Ascunce et al. [17]. A sequence of a closely related microsporidium *Kneallhazia* sp. found in a thief ant, *Solenopsis carolinensis* (GenBank Accession number: GU173849), was used as an outgroup.

The MEGA version X [20] was used to estimate a matrix of pairwise differences among SSU rDNA sequences using uncorrected *p*-distances (proportion of nucleotide sites at which two sequences being compared are different) as well as the total number of differences. All positions containing gaps and missing data were eliminated (complete deletion option), which led to a total of 760 positions in the final dataset. Neighbor-joining (NJ) trees [21] were generated using the matrix of *p*-distances. Bootstrapping was performed using 500 pseudo-replications of the dataset. The genealogical relationships among microsporidian variants were also analyzed using the program TCS Version 1.13 [22].

## 3. Results

### 3.1. Prevalence of the Microsporidium and the Social Parasite

The social parasite *S. daguerrei* was found in 180 nests out of the 219 *S. richteri* nests included in this study representing an average 82% of prevalence. *S. daguerrei* was found in every sampled *S. richteri* nest at four sites, and in 12 out of the 51 *S. richteri* nests.

The *K. solenopsae* SSU rRNA was successfully PCR amplified from DNA extractions of both ant species, *S. richteri* and its social parasite, *S. daguerrei*, indicating that both species harbor this microsporidium. Our PCR assay revealed that the microsporidian prevalence varied among sites and species. For *S. richteri*, all five sampled sites had some positive colonies, and 28 (12.8%) out of the 219 *S. richteri* colonies analyzed were positive for *K. solenopsae* (Table 1). For *S. daguerrei*, among the 180 inquiline social parasites found in *S. richteri* colonies, seven ants (3.9%) tested positive for *K. solenopsae* at only three out of the five sites. This is the first population prevalence report of *K. solenopsae* in *S. daguerrei* (Table 1). We observed SSU rRNA PCR-positive amplifications in *S. daguerrei* in all but one SSU rRNA PCR-positive *S. richteri* nest. This is that there was one SSU rRNA PCR-positive amplification from a *S. daguerrei* ant that was found in a S. richteri nest that had a negative PCR (no amplification) result.

### 3.2. Phylogenetic Results

**Figure 1:**
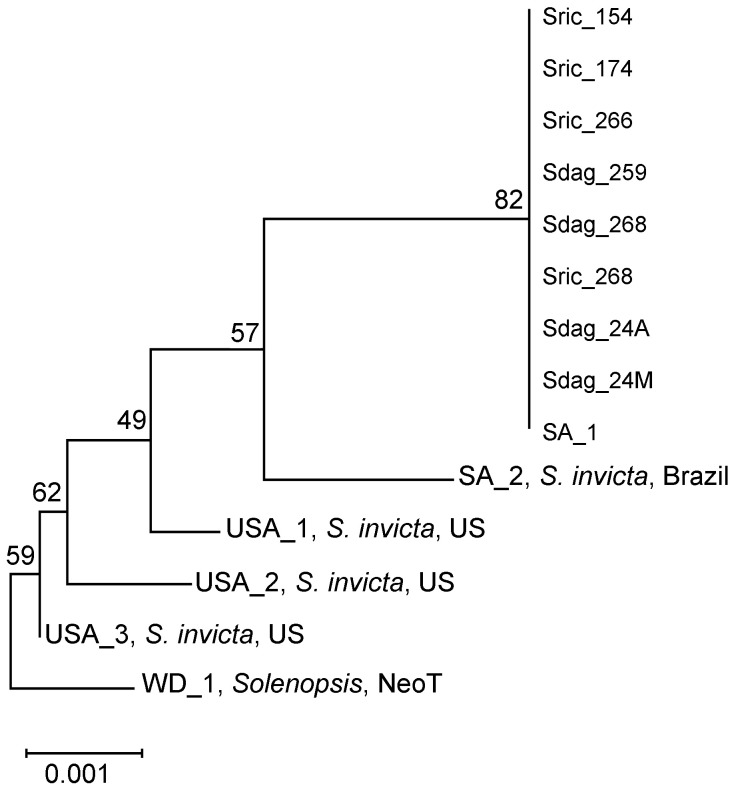
Neighbor-joining (NJ) tree constructed based on the *K. solenopsae* SSU rRNA *p*-distances found among fire ants. Numbers on branches represent bootstrap support values. Tree based on sequences recovered from *K. solenopsae* SSU rRNA found among *S. invicta* genotypes: SA_2, USA_1, USA_2, and USA_3, in bold are the *K. solenopsae* variants found among *S. richteri*: SA_1, Sric_154, Sric_174, Sric_266, and Sric_268, and italicized are the ones found among *S. daguerrei* ants: Sdag_259, Sdag_268, Sdag_24A and Sdag_24M. This tree was rooted using the WD_1 sequence, which is found in both *S. invicta* from the USA, as well as *S. geminata* from southern Mexico, and it belongs to the Neotropical (NeoT) *Kneallhazia* clade [17].

We obtained for the first time the sequence of the *K. solenopsae* SSU rRNA gene from the social parasite *S. daguerrei* (Table 2). However, further studies are needed to assess whether the microsporidium causes any type of negative effect on *S. daguerrei*. Based on the 760 bp alignment, which excluded gaps and missing data, all the *K. solenopsae* sequences found in *S. richteri* (host) and *S. daguerrei* (social parasite) were identical, showing zero differences (Table 3). Furthermore, the sequences were the same as the previously published *K. solenopsae* found in the *S. richteri* host (GenBank Accession number: AF031537, Moser et al. [16]), classified as Genotype SA_1 per Ascunce et al. [17]. Phylogenetic analyses using the *p*-distances revealed that the *K. solenopsae* variant found in *S. richteri* and *S. daguerrei* is closely related to the variants found in *S. invicta* from U.S. and South America (Figure 1 and Figure S1).

**Figure 2:**
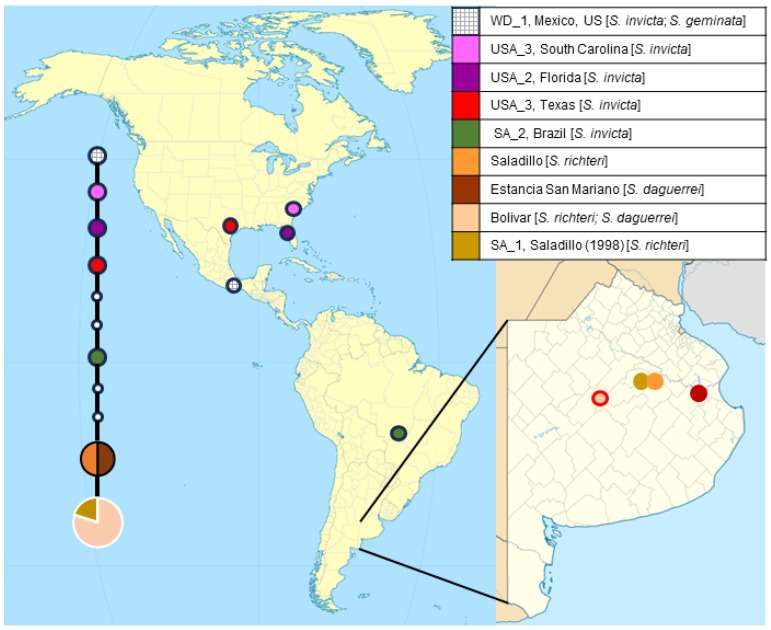
The statistical parsimony network and a map showing the distribution of *K. solenopsae* SSU rRNA variants throughout the Americas indicated by the colored pies. In the network, each connecting branch represents a single mutational step, and inferred missing intermediate haplotypes are represented by white circles. Sizes are scaled and represent relative frequencies. The legend explaining each of the colored pies including the host species, in brackets, is shown in the top right corner of the figure. A detailed of the Province of Buenos Aires, Argentina is shown in the bottom right corner of the figure.

We constructed a haplotype network using the statistical parsimony method implemented in the program TCS to further understand the relationships among the SSU rRNA sequences (Figure 2). The network method used in this study is based on statistical parsimony of the genealogical relationships among sequences and it included missing and ambiguous data. This network showed that the variant found in *S. richteri* (host) and *S. daguerrei* (social parasite) in one of the sites (Bolivar) matched the SA_1 genotype and was differentiated by one mutational step from the sequences obtained from the ants in the other geographic sites (Figure 2). Four of the new SSU rRNA sequences obtained in this study from *S. richteri* and *S. daguerrei* from Bolivar are shown in the same node with the SSU rRNA *K. solenopsae* variant obtained by Moser et al. [16] in *S. richteri* from Buenos Aires province. The sequence SA_2 corresponded to the SSU rRNA K. solenopsae variant found in *S. invicta* from Brazil (native range), and it was connected to the new sequences by three mutational steps. Three, four, and five mutational steps connected SA_2 to the USA_1, USA_2, and USA_3 variants found in *S. invicta* in the US, respectively. The variant WD_1 that was detected in both *S. invicta* (US) and *S. geminata* (Mexico) was the most distant from the new sequences and separated by nine mutational steps. There is a need to improve the resolution of the phylogenetic relationships among these variants by sequencing additional genes. A map with the sampling locations and the geographic distribution of the SSU rRNA *K. solenopsae* variants is presented in Figure 2.

## 4. Discussion

In this study, we surveyed the fire ant *S. richteri* and its inquiline social parasite *S. daguerrei* for the presence of the microsporidium *K. solenopsae*. Although the presence of *K. solenopsae* in these species has been reported previously [23], this is the first population prevalence report of this microsporidium in *S. daguerrei*. This is also the first attempt at characterizing the genetic diversity in this microsporidium among several *S. richteri* nests from different geographic sites and the microsporidian variants found in *S. daguerrei*. These SSU rRNA sequence data revealed that (a) *K. solenopsae* found in *S. richteri* is very similar (only one mutational step difference detected) among these populations located in Buenos Aires Province, Argentina; (b) *K. solenopsae* found in the inquiline social parasites were the same as their hosts; and (c) This microsporidian SSU rRNA variant is different from those found among *S. invicta* populations. Thus, our initial hypothesis and expectation were corroborated based on the current data.

### 4.1. Prevalence

Our survey data showed that *K. solenopsae* was present in all sites in *S. richteri*, with a prevalence variation from 5% to 23% of the colonies. The microsporidium was absent in some sites in the social parasite *S. daguerrei* analyzed, varying from 2% to 13% of the ants in other sites (Table 2). A previous long-term study in one of the sites (Saladillo) found a prevalence of *K. solenopsae* in *S. richteri* ranging from 22.4% at the beginning of the study to a final prevalence of 35.7% at the end of the study [14]. Interestingly, in this current study, Saladillo had the highest prevalence of *K. solenopsae* at 19.5%, which is close to one of the values found in the previous study [14]. Two differences between that study and this one are the assay used and the number of ants screened; Briano et al. [14] used thousands of ants per nest and detected the microsporidium via microscopy, whereas in our study, we only used 10 ants per nest and conducted the screening using PCR techniques. In another study, Briano et al. [24] also studied the prevalence of *K. solenopsae* among fire ants in Buenos Aires Province using microscopy. The authors found that some sites had prevalence values below 4%, with a highest value of 27% in one site and an overall average prevalence of 8% [24]. Our study builds upon prior research that suggests that *K. solenopsae* is a common pathogen of fire ants in Buenos Aires Province.

### 4.2. Genetic Identities of Microsporidian Variants

Sequence analyses of the host (*S. richteri*) and the social parasite (*S. daguerrei*) showed that both ant species share the same microsporidian variant per geographic site. We hypothesize that due to the higher prevalence of *K. solenopsae* in *S. richteri*, this ant is the native host of this microsporidium. In addition, the presence of the same variant in the two studied species likely results from horizontal transfer due to the close relationship between *S. richteri* and *S. daguerrei*, such that the pathogen *K. solenopsae* can be transmitted to *S. daguerrei*. The ability of this microsporidium to switch among *Solenopsis* host species has been previously suggested [17]. Host switching events have also been observed among the microsporidium *Nosema* infecting different species of bumblebees [25] and honey bees [26]. Interestingly, *Nosema ceranae* appears to have switched hosts from its native Asian honey bee (*Apis cerana*) host to the western honey bee (*Apis mellifera*) in areas where both honey bee species co-occur. In this new host, *N. ceranae* has a greater virulence than its congener *N. apis* [26]. Thus, it is possible that the lower prevalence of *K. solenopsae* in *S. daguerrei* could be due to a higher pathogenicity of this microsporidium in this non-native host. However, Briano et al. [15] presented a different interpretation and considered that the low number of *S. daguerrei* ants that carried *K. solenopsae* spores may suggest that *S. daguerrei* ants have merely ingested spores and were not a real host for the pathogen. Further studies are needed to understand the horizontal transmission patterns of this microsporidium within and among different hosts within a nest and the pathogenicity of *K. solenopsae* towards *S. daguerrei*.

### 4.3. Genetic Diversity

The SSU rDNA sequences from *S. richteri* and *S. daguerrei* were identical per geographic site. Among all the SSU rRNA sequences from the NeoT clade the percent of pairwise *p*-distances varied from 0.11 to 0.4% (Table 2). Thus, additional gene sequence data are needed to resolve the phylogenetic relationships among *K. solenopsae* variants found among the different *Solenopsis* species, as was previously suggested [17]. There is a pressing need to characterize the genome of *K. solenopsae* as an initial step in unraveling the many aspects of *K. solenopsae*-fire ant evolutionary interactions. The study of coding genes will also open the door to functional transcriptomic studies to gain a deeper understanding of pathogenicity and transmission modes.

## 5. Conclusions

Further studies are needed to gain a better understanding of the evolutionary history of *K. solenopsae* among fire ants in their native range in South America, as well in their invasive range in the U.S. Gaining a deeper understanding of the genetics and biology of this microsporidium species is crucial for developing effective biological control methods for invasive populations of *S. invicta*, *S. richteri,* and its hybrids in the U.S. Determining the pathogenicity (including infectivity and virulence) of genetically different *K. solenopsae* variants could lead to discovery of new variants that are more effective in controlling fire ants in the U.S. This tri-partite system with the microsporidium *Kneallhazia*, *S. richteri,* and its social parasitic ant provides an ideal model for studying the epidemiological dynamics of the microsporidium within a colony. Such knowledge can help to understand the origin, reservoirs, and transmission of this microsporidium and possibly other ant microsporidium species.

## Figures and Tables

**Table 1:** Prevalence of *Kneallhazia solenopsae* in *S. richteri* and its inquiline social parasite, *S. daguerrei*, in Buenos Aires Province, Argentina. For *S. richteri*, a bulk sample of 10 ants per nest was analyzed. For those *S. richteri* nests where *S. daguerrei* was found, one *S. daguerrei* ant per *S. richteri* nest was screened for the presence of *K. solenopsae*.

	*S. richteri*	*S. daguerrei*
Locality	% infected nests	% infected ants
(latitude, longitude)	(# of infected/total nests)	(# of infected/total ants)
Mercedes	5.1	2.6
(34°39′ S, 59°26′ W)	(2/39)	(1/39)
Suipacha	11.8	0.0
(34°46′ S, 59°41′ W)	(6/51)	(0/12)
Saladillo	19.5	0.0
(35°38′ S, 59°46′ W)	(8/41)	(0/41)
Bolivar	22.5	12.5
(36°15′ S, 61°06′ W)	(9/40)	(5/40)
Estancia San Mariano	6.3	2.1
(36°4’ S, 57°51’ W)	(3/48)	(1/48)
Total	12.8	3.9
	(28/219)	(7/180)

Note: # means number.

**Table 2:** Information of *K. solenopsae* SSU rRNA sequences, their ant host species, and GenBank accession number information.

Sequence	Host/s	Geographical Distribution/Origin	Major	GenBank
Name			Lineage	Accession Number
WD_1	*S. invicta*	Mexico, Florida, South Carolina	NeoT	HM026467
	*S. geminata*			
USA_1	*S. invicta*	Texas	NeoT	HM026464
USA_2	*S. invicta*	Florida	NeoT	HM026465
USA_3	*S. invicta*	South Carolina	NeoT	HM026466
SA_2	*S. invicta*	Mato Grosso, Brazil	NeoT	AF031538
SA_1	*S. richteri*	Buenos Aires, Argentina	NeoT	AF031537
Sric_154	*S. richteri*	Saladillo, Buenos Aires, Argentina	NeoT	OR519910
Sric_174	*S. richteri*	Saladillo, Buenos Aires, Argentina	NeoT	OR519911
Sric_266	*S. richteri*	Bolivar, Buenos Aires, Argentina	NeoT	OR519912
Sdag_259	*S. daguerrei*	Bolivar, Buenos Aires, Argentina	NeoT	OR519913
Sdag_268	*S. daguerrei*	Bolivar, Buenos Aires, Argentina	NeoT	OR519914
Sric_268	*S. richteri*	Bolivar, Buenos Aires, Argentina	NeoT	OR519915
Sdag_24A	*S. daguerrei*	Estancia San Mariano, Buenos Aires, Argentina	NeoT	OR519916
Sdag_24AM	*S. daguerrei*	Estancia San Mariano, Buenos Aires, Argentina	NeoT	OR519917

**Table 3:** Matrix of pairwise *p*-distances (above diagonal) and number of differences (below the diagonal) among *K. solenopsae* SSU rRNA sequences found in *S. invicta* (SA_2, WD_1, USA_1, USA_2, USA_3), among *S. richteri* (SA_1, Sric_154, Sric_174, Sric_266, Sric_268, names in bold), and among *S. daguerrei* ants (Sdag_259, Sdag_268, Sdag_24A and Sdag_24M, names in italics).

	WD_1	USA_1	USA_2	USA_3	SA_2	**SA_1**	**Sric_154**	**Sric_174**	**Sric_266**	*Sdag_259*	*Sdag_268*	**Sric_268**	*Sdag_24A*	*Sdag_24M*
WD_1		0.003	0.003	0.001	0.004	0.003	0.003	0.003	0.003	0.003	0.003	**0.003**	0.003	0.003
USA_1	2		0.000	0.001	0.001	0.003	0.003	0.003	0.003	0.003	0.003	0.003	0.003	0.003
USA_2	2	0		0.001	0.001	0.003	0.003	0.003	0.003	0.003	0.003	0.003	0.003	0.003
USA_3	1	1	1		0.003	0.001	0.001	0.001	0.001	0.001	0.001	0.001	0.001	0.001
SA_2	3	1	1	2		0.004	0.004	0.004	0.004	0.004	0.004	0.004	0.004	0.004
**SA_1**	2	2	2	1	3		0.000	0.000	0.000	0.000	0.000	0.000	0.000	0.000
**Sric_154**	2	2	2	1	3	0		0.000	0.000	0.000	0.000	0.000	0.000	0.000
**Sric_174**	2	2	2	1	3	0	0		0.000	0.000	0.000	0.000	0.000	0.000
**Sric_266**	2	2	2	1	3	0	0	0		0.000	0.000	0.000	0.000	0.000
*Sdag_259*	2	2	2	1	3	0	0	0	0		0.000	0.000	0.000	0.000
*Sdag_268*	2	2	2	1	3	0	0	0	0	0		0.000	0.000	0.000
**Sric_268**	2	2	2	1	3	0	0	0	0	0	0		0.000	0.000
*Sdag_24A*	2	2	2	1	3	0	0	0	0	0	0	0		0.000
*Sdag_24M*	2	2	2	1	3	0	0	0	0	0	0	0	0	

## Data Availability

All data from this research has been included in this manuscript and sequences have been deposit in GenBank (GenBank Accession numbers OR519910 to OR519917).

## Supplementary Materials

The following supporting information can be downloaded at: https://www.mdpi.com/article/10.3390/insects14120901/s1, Figure S1: Neighbor-joining (NJ) tree constructed based on *p*-distances based on *K. solenopsae* SSU rRNA gene sequences found among fire ants. This tree was rooted using *Kneallhazia* sp. found in a thief ant, *Solenopsis carolinensis*, as an outgroup.
