# Supplementary material for: Prevalence and Genetic Diversity of a Microsporidian Parasite in the Black Imported Fire Ant and Its Social Parasitic Ant (Formicidae: Myrmicinae: Solenopsis) in Buenos Aires Province, Argentina
Source: Insects. 2023 Nov 21;14(12):901. doi: 10.3390/insects14120901 (PMC10743500; doi:10.3390/insects14120901)
Supplement: Supplementary file 1 [file insects-14-00901-s001.zip › insects-2641158-supplementary.pdf]

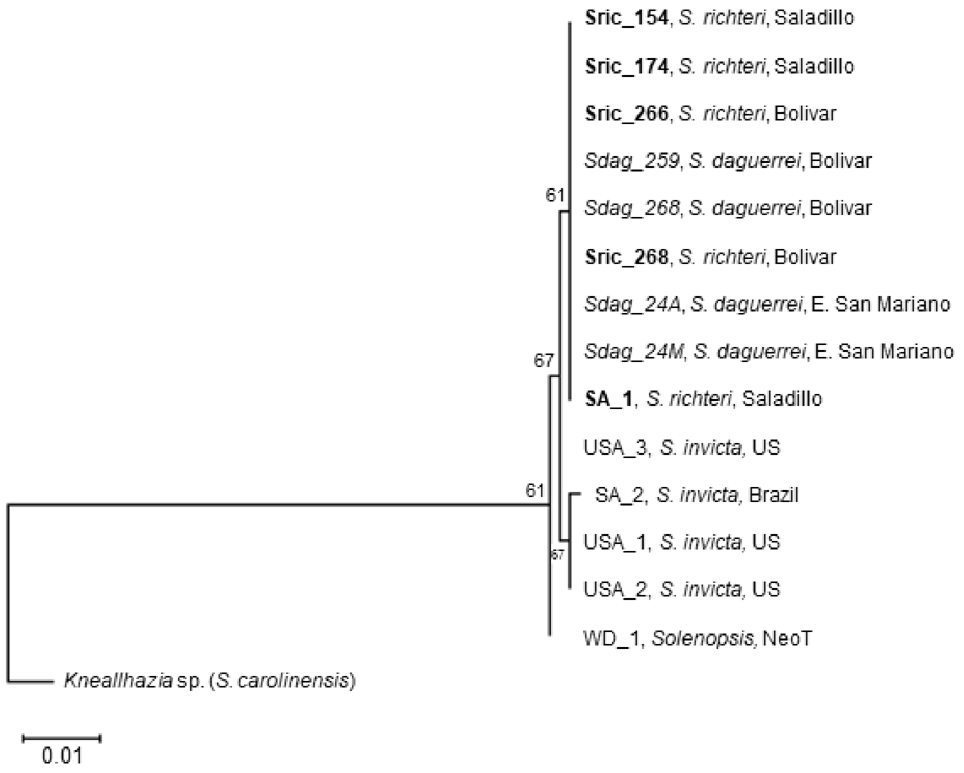

Figure S1. Neighbor-joining (NJ) tree constructed based on p-distances based on *K. solenopsae* SSU rRNA gene sequences found among fire ants. Numbers on branches represent bootstrap support values. Tree based on sequences recovered from *K. solenopsae* 16S rRNA found among *S. invicta* genotypes: SA_2, WD_1, USA_1, USA_2, and USA_3, in bold are the *K. solenopsae* variants found among *S. richteri*: SA_1, Sric_154, Sric_174, Sric_266, and Sric_268, and italicized are the ones found among *S. daguerrei* ants: Sdag_259, Sdag_268, Sdag_24A and Sdag_24M. This tree was rooted using *Kneallhazia* sp. found in a thief ant, *Solenopsis carolinensis*, as an outgroup.
